# Close Call After a Fall: Understanding Traumatic and Atraumatic Splenic Rupture and Its Life-Threatening Consequences

**DOI:** 10.7759/cureus.45544

**Published:** 2023-09-19

**Authors:** Cheri L Lotfi, Charles E Adams, Reginald Saint-Hilaire

**Affiliations:** 1 Anesthesiology, Lake Erie College of Osteopathic Medicine, Bradenton, USA; 2 Osteopathic Medicine, Lake Erie College of Osteopathic Medicine, Bradenton, USA; 3 Emergency Medicine, Tampa General Hospital, Tampa, USA

**Keywords:** hyperlipidemia, hypertension, aeromed, emergency department, atraumatic splenic rupture, traumatic splenic rupture, fall injury, splenic laceration, splenic trauma

## Abstract

Splenic rupture, a critical surgical emergency involving the tearing of the spleen's capsule and the ensuing internal bleeding, primarily results from abdominal trauma or underlying medical conditions affecting the spleen. A 71-year-old male with hypertension and hyperlipidemia suffered a mechanical fall, leading to his presentation in the emergency department. Despite a stable initial condition and discharge, he returned the following day with dizziness and severe anemia. Subsequent diagnostics revealed a ruptured spleen, necessitating immediate surgical intervention. This case emphasizes traumatic and atraumatic causes of splenic rupture, with older adults, anticoagulant users, and viral illnesses accentuating vulnerability. Physical exam findings might be absent, highlighting the importance of considering splenic rupture in cases of unexplained hemodynamic instability. In this instance, a combination of trauma, a possible history of anticoagulation use, and a recent viral illness contributed to the patient's splenic rupture. The case underscores the need to retain a high index of suspicion for splenic rupture even without obvious physical findings, advocating for diligent evaluation of abnormal vital signs.

## Introduction

Splenic rupture refers to the disruption of the splenic capsule or tearing apart of the spleen, a vital organ responsible for filtering blood and fighting infections. This condition is considered a surgical emergency, as it can cause significant and potentially life-threatening internal bleeding [1]. Splenic rupture occurs primarily as a result of blunt trauma to the abdomen such as from a car accident or a fall. Certain medical conditions that affect the spleen, including mononucleosis (EBV), leukemia, or lymphoma are also less common but important causes of splenic rupture [1].

## Case presentation

A 71-year-old male with a history of hypertension and hyperlipidemia was brought to the emergency department (ED) by his wife after experiencing a mechanical fall at home. The patient had been recovering from a recent viral illness and had been experiencing balance issues. He had been taking Eliquis for the past year due to an episode of atrial fibrillation in addition to his antihypertensive medication. His Glasgow Coma Scale was 15 at the time of arrival at the ED. When questioned, the patient denied losing consciousness during the fall. The physical exam revealed an elderly gentleman in acute distress with a 4-cm laceration noted to the left temporal scalp. No depressed skull fracture was noted. The exam was otherwise within normal limits. The patient's vitals were heart rate (HR) 74 bpm, respiratory rate (RR) 20/min, BP 94/62 mmHg, and O_2_ Sat. 95%. An ECG showed normal sinus rhythm without ectopic or ischemic changes, and a viral panel was positive for Influenza A. A complete blood count (CBC) revealed a low hemoglobin of 10.7 g/dl and hematocrit of 32.8%. A CT of the head without contrast was performed to rule out cerebral hemorrhage, and there was no acute intracranial abnormality noted. The patient received staples for his scalp laceration and was discharged from the ED.

Nine hours later, however, the patient returned to the ED complaining of dizziness and lightheadedness that intensified with any change in body position. The only abnormality on the patient’s physical exam at this time was the pallor of his skin. Although he had a regular rate and rhythm, he had a worrisome blood pressure of 69/48 mmHg. He was given a 250 mL normal saline fluid bolus and a CBC, complete metabolic panel (CMP), and troponin were ordered. Two serial ECGs both showed normal sinus rhythm with no immediate ST-T changes and the troponin levels came back within normal limits. However, the CMP results showed that the patient's blood urea nitrogen (BUN) and creatinine (Cr) levels were elevated. On the review of the repeat CBC, the patient's hemoglobin levels had plummeted to 6.0 g/dl and hematocrit to 19.2%.

**Figure 1:**
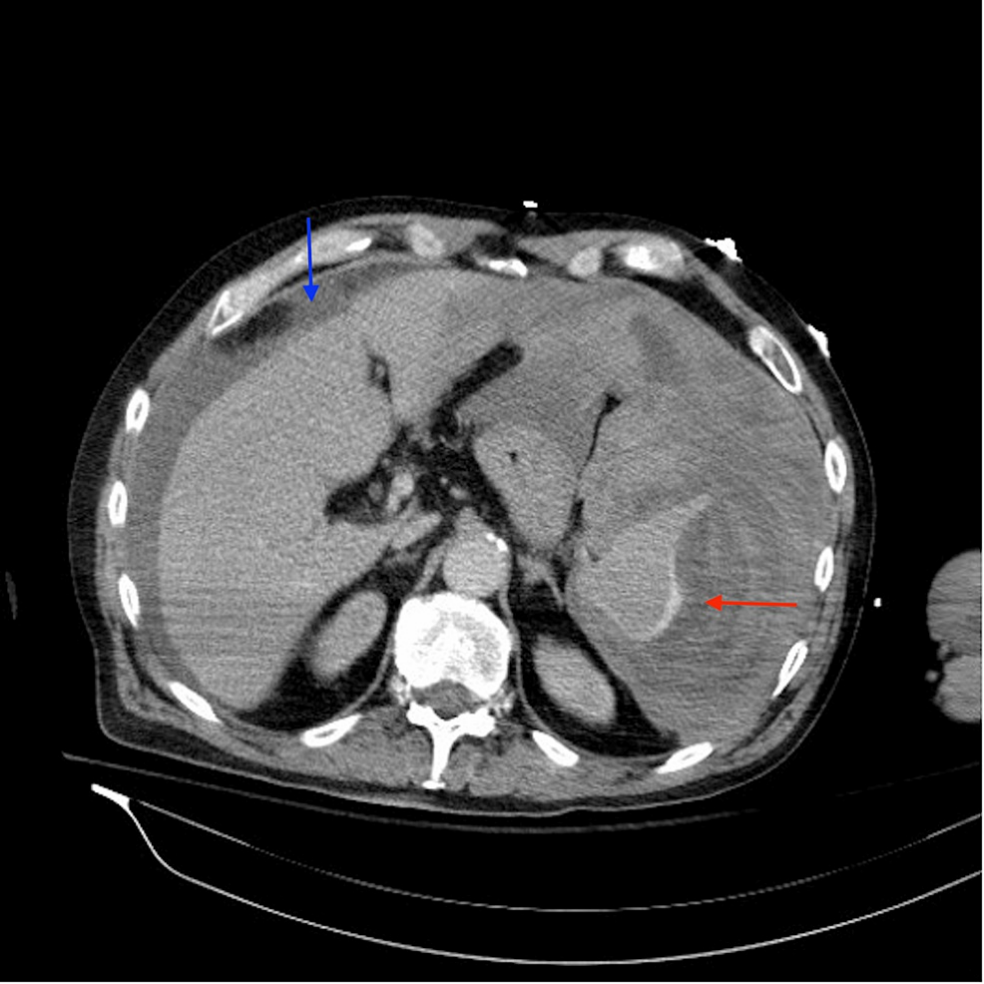
CT abdomen with contrast CT abdomen with contrast revealing a large subcapsular hematoma compressing the spleen (red arrow), with mild-to-moderate serosanguineous ascites (blue arrow). Subcapsular venous extravasation is noted under the lateral splenic capsule.

**Figure 2:**
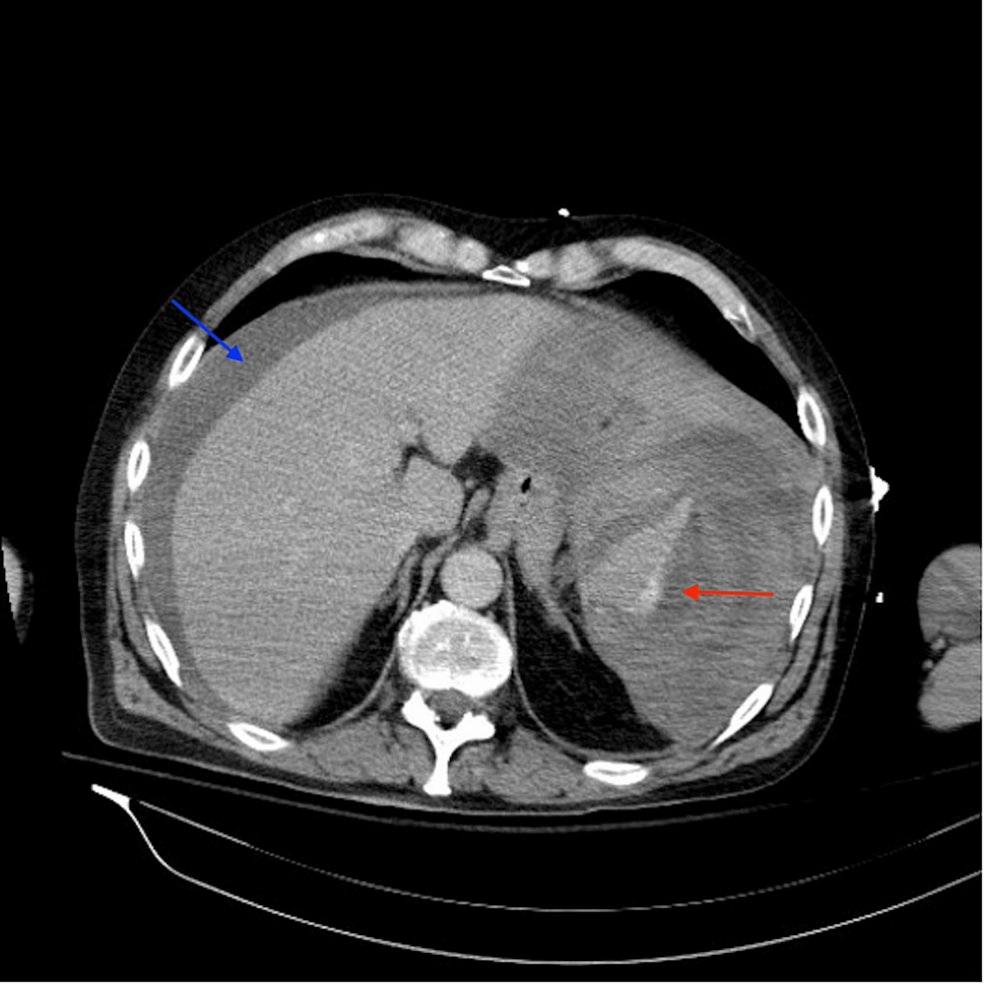
CT abdomen with contrast CT abdomen with contrast revealing a large subcapsular hematoma compressing the spleen (red arrow), with mild-to-moderate serosanguineous ascites (blue arrow). Subcapsular venous extravasation is noted under the lateral splenic capsule.

The emergency medicine physician immediately ordered a CT scan of the chest and abdomen with contrast to pinpoint the source of the bleeding. Soon thereafter, the physician received a call from the radiologist who informed him that the patient had a ruptured spleen. The CT scan of the abdomen revealed a large subcapsular hematoma compressing the spleen, with mild-to-moderate serosanguineous ascites (Figure 1). Subcapsular venous extravasation was noted under the lateral splenic capsule (Figure 2). To stabilize the patient, the team sent a type and crossmatch and administered four units of blood before transferring him via Aeromed for immediate surgical intervention.

## Discussion

Splenic rupture is generally categorized into traumatic and atraumatic causes. Traumatic splenic rupture typically results from blunt abdominal trauma, including compressive forces against the abdomen or shearing forces created by sudden deceleration [2]. The most susceptible patients are older adults, alcoholic patients, and in the case of motor vehicle accidents, those wearing safety belts without shoulder attachments [3]. Lacerations of the spleen can occur from fractured ribs or pelvic bones, and a history of trauma to the left upper quadrant, left rib cage, or left flank should increase suspicion of splenic injury [4]. Patients with traumatic splenic rupture may present with left upper abdominal, left chest wall, or left shoulder pain from diaphragm irritation, also known as Kehr's sign [3,5]. Other physical manifestations include left upper quadrant or generalized abdominal tenderness, abdominal wall contusion or hematoma (e.g., seat belt sign), as well as left lower chest wall tenderness, contusion, or instability due to rib fractures [3].

Atraumatic splenic rupture (ASR) is less common but can occur in a wide age range, from teenagers to the elderly. The majority of cases are "pathologic" ASRs, meaning they develop in a diseased spleen due to infection, coagulopathy, or neoplasm [6]. In some cases, ASR represents the initial manifestation of an underlying disease. Alternatively, "idiopathic" ASR can occur in a normal-appearing spleen without predisposing factors. Anticoagulation is a common cause of idiopathic ASR, accounting for up to 9% of cases [6]. Patients with ASR may present with variable degrees of upper or left-sided abdominal pain, tachycardia, and hypotension, followed by malaise, vomiting, generalized abdominal tenderness, peritonitis, and progressive hemodynamic shock [7].

In the case of this patient, there were many variables that likely contributed to his splenic rupture: his fall, anticoagulation therapy, and a recent history of influenza A. Key details surrounding the nature and mechanism of this patient’s fall remain limited such as his position at the time of the incident, the height of the fall, and what triggered the fall. On his initial presentation, the patient's hypotension and history of a recent fall did raise suspicion for another injury apart from his scalp laceration, however, he denied any abdominal pain on physical exam. His past medical history of atrial fibrillation and anticoagulation with Eliquis increased his risk for spontaneous bleeding and theoretically for atraumatic splenic rupture. Similarly, his recent viral illness could have played a role in causing ASR as well due to an increase in intrasplenic tension from cellular hyperplasia and vascular engorgement [8].

**Figure 3:**
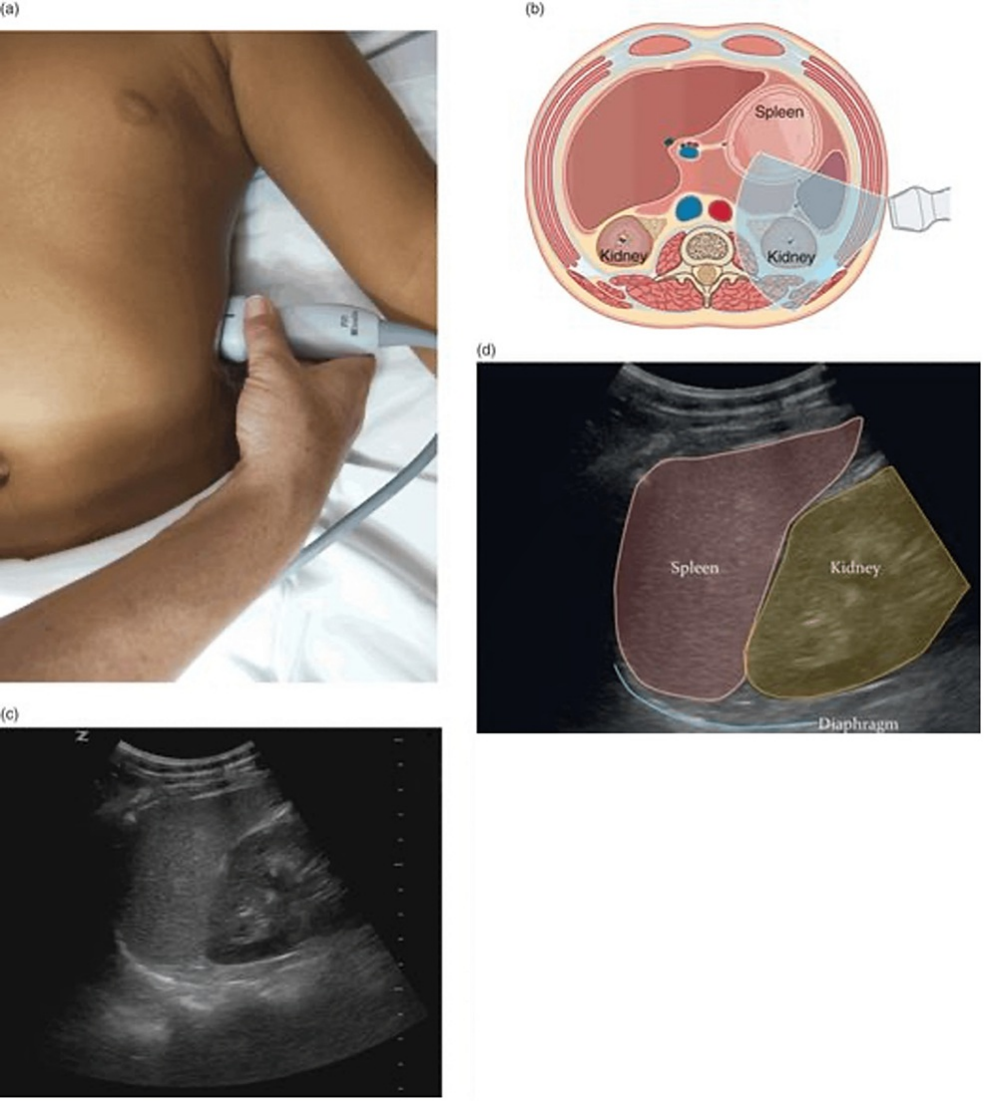
Left upper quadrant FAST Left upper quadrant (splenorenal) view. (a) Transducer placement at the left upper quadrant (LUQ). (b) Illustration of transducer placement and relevant anatomy. Artwork created by Emily Evans © Cambridge University Press. (c) Normal ultrasound image of the splenorenal space or left upper quadrant. (d) Illustration of important structures visualized in the ultrasound image including the kidney, diaphragm, and spleen. Illustration by Laura Berg, MD [9]. DISCLAIMER: The images provided in Figure 3 are not the FAST images from our patient, but rather a demonstration of a negative FAST in the LUQ. Written permission from the original publishers was obtained to reproduce these images. FAST: Focused Assessment with Sonography in Trauma

**Figure 4:**
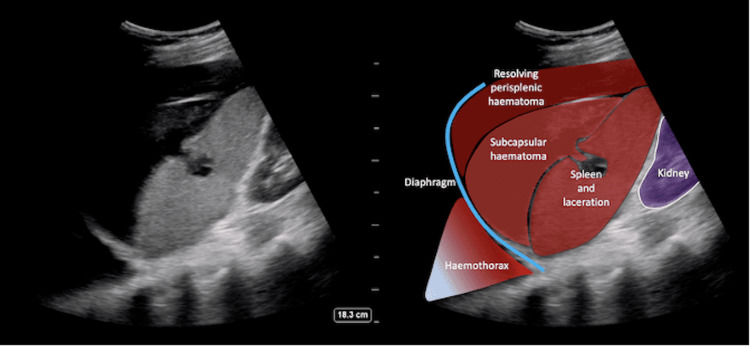
Splenic rupture FAST FAST left upper quadrant (LUQ) view showing that there is heterogeneous predominantly hyperechoic perisplenic hematoma as well as subcapsular hematoma. The echogenic area in the mid portion of the spleen disturbing the usual homogeneous echotexture suggests a laceration [10]. DISCLAIMER: The FAST images provided are not the FAST images from our patient, but rather a positive FAST in the LUQ. Written permission from the original publishers was obtained to reproduce these images. FAST: Focused Assessment with Sonography in Trauma

While the exact cause of this patient’s splenic rupture was most likely the combination of his anticoagulation therapy and his fall, this case is presented as a noteworthy example of how splenic rupture can occur in the absence of overt physical exam findings. On physical exam, the patient had negative Kehr's sign and no abdominal pain or rebound tenderness to palpation. This demonstrates that splenic rupture cannot be excluded from the differential due to an unremarkable physical examination in the appropriate clinical scenario. A Focused Assessment with Sonography in Trauma (FAST) was not deemed to be warranted on his initial presentation due to the absence of any positive physical exam findings; however, perhaps his hypotension should have prompted further pursuit of an explanation apart from the fact that he took his blood pressure medications earlier that morning (Figure 3, Figure 4). Nonetheless, the FAST exam is a critical diagnostic tool that should always be considered in a patient presenting with hypotension with or without low hemoglobin.

## Conclusions

In summary, splenic rupture is generally classified into two categories: traumatic and atraumatic. The former is typically the result of blunt abdominal trauma while the latter can occur due to a variety of pathologic causes such as infection, malignancy, and systemic anticoagulation. This patient’s case of splenic rupture is interesting, as he not only had a history of trauma and anticoagulation use but also had a recent history of viral illness. This case also serves as a sobering reminder that even in the absence of any remarkable findings on a physical abdominal exam, it is essential for emergency medicine physicians to keep the possibility of splenic rupture in mind and consider performing a FAST in the setting of unexplained abnormal vital signs.
